# Investigating Medical Ideas Transmitted Across Traditional Education: A Pilot Study

**DOI:** 10.7759/cureus.78125

**Published:** 2025-01-28

**Authors:** Joshua Moen, Chloe Shuck

**Affiliations:** 1 School of Community Medicine, University of Oklahoma Health Sciences Center, Tulsa, USA

**Keywords:** alternative system of medicine, critical theory, higher education medical training, learning pedagogy and teaching, social-ecological framework

## Abstract

Introduction

Deep-rooted beliefs in medical education may hinder innovation and optimal patient care by perpetuating existing paradigms. This pilot study examined how medical beliefs are distributed among different professional groups within a single academic medical center, reflecting the influence of cultural and educational reproduction.

Methods

We conducted a small cross-sectional survey from August to December 2024 at a single medical teaching institution. Participants were physicians (n = 21), physician assistant (PA) faculty (n = 4), residents (n = 11), medical students (n = 38), and PA students (n = 41). A ten-question survey assessed beliefs about medical knowledge sources, sleep practices, biomarker utility, medication effectiveness, disease progression, and dietary interventions. Data were analyzed using chi-square tests and one-way ANOVA, with significance set at p < 0.05.

Results

Of 115 respondents (response rate ~39-46%), significant associations were found between professional role and gender distribution (χ² = 12.6, p = 0.013) and between professional role and primary source of medical knowledge (χ² = 28.8, p = 0.004). Practicing clinicians relied exclusively on standardized content, while students and residents utilized more diverse sources. Although differences in medical beliefs across roles were not statistically significant, patterns indicated reinforcement of existing paradigms and potential constraints on critical thinking.

Conclusion

The findings suggest that medical education may perpetuate entrenched beliefs through reliance on standardized content, potentially limiting critical thinking and innovation. The emphasis on standardized testing and content mastery may discourage the exploration of alternative perspectives. These results highlight the need for medical education to foster critical thinking and incorporate new evidence to better prepare adaptable healthcare professionals.

## Introduction

Entrenched beliefs and practices within the medical community can persist despite advances in evidence-based medicine, potentially hindering innovation and optimal patient care. Cultural and educational reproduction, a concept rooted in the sociological work of Pierre Bourdieu [1], suggests that educational institutions often perpetuate existing social structures and belief systems. Bourdieu's theory of reproduction posits that these social structures and belief systems are preserved by transmitting the "cultural capital" of dominant groups, thereby reinforcing the status quo. In medical education, this may manifest as the reinforcement of specific beliefs about health, disease, and treatment approaches across different levels of the medical hierarchy. This process can contribute to the persistence of outdated or unsupported practices within the profession [2,3].

Despite this understanding, there is a gap in knowledge regarding how these medical beliefs are distributed among various groups within medical institutions, such as physicians, physician assistants (PAs), medical residents, medical students, and PA students. This lack of insight hinders the identification of discrepancies between current evidence-based practices and the beliefs held by these groups, potentially revealing areas where resistance to change exists within the medical culture. To address this gap, our study aimed to investigate how established medical beliefs are distributed across different professional roles within a medical institution and how these beliefs may reflect the processes of cultural and educational reproduction described by Bourdieu. Specifically, we sought to determine whether medical beliefs regarding fundamental health concepts differed among physicians, PAs, residents, medical students, and PA students and whether these differences suggested a pattern of belief transmission consistent with Bourdieu's theory. Additionally, we aimed to identify beliefs that persist across professional levels despite contradicting current evidence-based practices, which may indicate systemic resistance to change.

To explore these issues, we conducted a cross-sectional pilot study at the University of Oklahoma - Tulsa School of Community Medicine. By investigating beliefs about fundamental health concepts such as sleep, biomarkers, medication effectiveness, chronic disease management, and dietary recommendations, we aimed to uncover patterns reflecting the influence of cultural and educational reproduction. Through critical analysis of the collected data, this study sought to illuminate the mechanisms by which beliefs persist in medical education and practice, ultimately contributing to efforts to enhance medical training and patient care outcomes.

## Materials and methods

Study design and setting

A cross-sectional survey was conducted at the University of Oklahoma - Tulsa School of Community Medicine, Tulsa, between August and December 2024. The study examined beliefs about key medical concepts across different levels of medical education and practice.

Participants

The study population included physicians (n = 21), PA faculty (n = 4), medical residents (n = 11), medical students (n = 38), and PA students (n = 41) from a single academic medical center. All current faculty, residents, and students at the institution were eligible for participation. From an estimated total eligible population of 250-295 individuals, we received 115 complete responses, representing a response rate of approximately 39-46%.

Survey development and content

The survey instrument was developed to explore areas where emerging evidence challenges established medical paradigms, aiming to identify potentially entrenched beliefs within medical education. The topics were derived through iterative discussions with 15 colleagues, including physicians, pharmacists, advanced practice providers, and students. These discussions revealed conflicting beliefs and knowledge in certain areas and highlighted topics that were frequently debated due to differing perspectives.

Building on these insights, a literature review was conducted to analyze the topics most commonly identified as sources of conflict. The key areas selected for the survey included research bias, sleep practices, biomarker utility, medication effectiveness, disease progression, and dietary interventions. Each topic was chosen for its relevance to conflicting medical paradigms and its potential to reflect how traditional beliefs persist despite new evidence.

Questions were then developed to assess the strength of beliefs in these areas of conflict. The survey utilized Likert and semantic differential scales to facilitate quantitative analysis while capturing nuances in participants' beliefs. For example, questions about sleep practices evaluated beliefs regarding the necessity of long work hours and sleep deprivation in medical training, despite evidence highlighting their negative impact on cognitive function and patient safety. Similarly, questions on chronic disease management assessed beliefs about the inevitability of disease progression and the reliance on medications versus lifestyle interventions.

Data collection

Survey distribution occurred through Qualtrics (Qualtrics, UT, USA), a secure web-based platform. Participants received an initial email invitation followed by two reminder emails at weekly intervals. The survey remained open for 16 weeks, with data collection occurring between August 15 and December 15, 2024. Basic demographic information included gender, current role, and professional experience level. All responses were anonymous and encrypted.

Statistical analysis

Data analysis was conducted using Qualtrics' native analytics tools. After excluding responses with more than 20% missing data, we performed descriptive statistics for all demographic variables and survey responses. For categorical variables, we calculated frequencies and percentages. For each survey question, we determined means, standard deviations, medians, and frequency distributions, both for the total sample and within each participant category (physicians, PA faculty, medical residents, medical students, and PA students). Chi-square analyses examined associations between professional roles and categorical response patterns. For comparing means across the five participant categories, we utilized Qualtrics' one-way analysis of variance (ANOVA) capabilities with subsequent pairwise comparisons. Effect sizes were calculated using Cramér's V for chi-square analyses and Cohen's f for ANOVA comparisons to determine practical significance beyond statistical significance. For all statistical tests, significance was set at p < 0.05.

Ethical considerations

The study received approval from the institutional review board (IRB #17659). Electronic informed consent was obtained from all participants before survey completion. Data were stored securely and analyzed in aggregate to protect confidentiality. Participation was voluntary, and no compensation was provided.

## Results

As the United States, in addition to many industrialized nations, continues to experience worsening health outcomes and decreased health spans while increasing expenditure, the need to look at the medical education system is imperative. Our results suggest that enculturation exists at the core of the medical education system in one of two entities, belief reinforcement or belief conception. Table 1 lists the raw counts of the survey responses for each survey question stratified by professional identity.

**Table 1:** Survey response by professional identity Raw counts of survey responses for each question, stratified by professional identity. Responses are grouped by professional roles: physician assistant (PA) students, medical students, medical residents, practicing physician assistants, and physicians. The table displays the number of respondents for gender distribution and each survey item, highlighting beliefs and perspectives on various medical education and practice topics across different professional groups.

	Physician Assistant Student	Medical Student	Medical Resident	Physician Assistant	Physician
Male	9	22	5	2	12
Female	32	16	6	2	9
What is your primary source of medical knowledge and information?					
Written, video, and/or audio content (e.g., textbooks, journal articles, educational videos, lectures, podcasts, audio lectures)	27	29	8	4	21
Peers (e.g., discussions with colleagues, study groups)	1	0	1	0	0
Mentors/faculty (e.g., direct instruction, clinical supervision)	12	9	0	0	0
Other	1	0	2	0	0
To what extent do you believe the "calories in, calories out" model is effective for weight management?					
Not at all sufficient 1	4	4	3	0	5
Somewhat sufficient 2	24	16	6	4	14
Moderately sufficient 3	8	13	2	0	1
Completely sufficient 4	1	1	0	0	0
How much do you agree that most chronic conditions are progressive and require lifelong medication?					
Strongly disagree 1	2	1	1	0	1
Somewhat disagree 2	16	10	2	0	4
Somewhat agree 3	20	22	6	3	9
Strongly agree 4	0	1	0	1	4
To what extent do you believe that evidence-based guidelines in medicine are free from bias?					
Highly biased 1	1	2	0	0	0
Moderately biased 2	25	21	3	1	6
Moderately biased 3	8	10	5	1	13
Highly unbiased 4	0	0	0	0	2
In your view, how significant is the impact of food additives and processing on overall health?					
Extremely significant 1	22	14	3	0	7
Moderately significant 2	15	14	5	4	7
Somewhat significant 3	2	6	0	0	4
Not significant 4	0	0	1	0	0
How strongly do you believe that achieving target biomarker levels (e.g., A1C, LDL) is a successful indicator in managing chronic conditions?					
Strongly agree 1	6	10	4	2	7
Somewhat agree 2	27	24	6	2	12
Neither agree nor disagree 3	0	0	0	0	0
Somewhat disagree 4	6	2	1	0	1
Strongly disagree 5	1	1	0	0	1
How likely do you think it is for a patient with type 2 diabetes to achieve remission without long-term medication use?					
Extremely likely 1	2	0	2	0	0
Somewhat likely 2	15	14	2	1	7
Somewhat unlikely 3	16	15	6	2	10
Extremely unlikely 4	4	4	1	1	4
In your opinion, how effective are medications compared to lifestyle changes in managing chronic diseases?					
Lifestyle changes are more effective 1	19	15	3	3	7
Lifestyle changes are slightly more effective 2	11	14	5	0	7
Medication is slightly more effective 3	9	6	2	0	3
Medication is more effective 4	0	0	0	1	2
To what extent do you believe that long work or study hours in place of sleep are necessary for becoming a competent medical professional?					
Extremely unnecessary 1	8	9	4	1	5
Somewhat unnecessary 2	13	14	3	2	6
Somewhat necessary 3	17	13	3	0	7
Extremely necessary 4	1	1	1	1	3
How confident are you in the current education system's ability to incorporate the latest research findings into clinical recommendations?					
Not at all confident 1	4	4	0	0	1
Somewhat confident 2	21	12	5	1	10
Moderately confident 3	9	15	4	3	6
Extremely confident 4	3	5	0	0	2

Utilizing chi-square and pairwise analyses to compare the beliefs between groups, two significant relationships emerged from our analyses. First, there was a significant association between professional role and gender distribution (χ2 = 12.6. , df = 4, p = 0.013, Cramér's V = 0.331). PA students showed the largest gender disparity, with 78.0% female (n = 32) and 22.0% (n = 9) male respondents, while medical students had a reversed ratio with 57.9% male (n = 22) and 42.1% female (n = 16). Practicing physicians were predominantly male (57.1%, n = 12), while residents and PAs showed equal gender distribution (50.0% each, n = 2).

Second, professional role was significantly associated with the primary source of medical knowledge (χ2 = 28.8, df = 12, p = 0.004, Cramér's V = 0.289). While written, video and audio content were the predominant sources across all groups, the reliance on secondary sources varied by professional level. PA students reported 65.9% (n = 27) usage of written/video/audio content and 29.3% reliance on mentors/faculty (n = 12). Medical students showed a similar pattern (76.3% written/video/audio, 23.7% mentors/faculty; n = 29 and 9, respectively). Both practicing physicians and PAs reported exclusively using written/video/audio content (100%; n = 25), while residents displayed the most diverse pattern (72.7% written/video/audio, 9.1% peers, 18.2% other sources; n = 8, 1, and 2, respectively).

The remainder of the statistical testing revealed non-significant differences between professional identity and medical beliefs. However, despite the lack of statistical significance, several noteworthy patterns emerged across professional roles regarding medical beliefs and practices. While not reaching traditional statistical significance (ranked ANOVA: p = 0.062, Cohen's f = 0.459), pairwise comparisons revealed meaningful differences in views on evidence-based guidelines' freedom from bias. Physicians rated guidelines as significantly less biased (mean = 2.81) compared to both medical students (mean = 2.24, p = 0.011) and PA students (mean = 2.21, p = 0.004). The effect sizes for these differences were large (effect sizes = -1.00 and 1.16, respectively). Residents and PAs showed intermediate values (means = 2.63 and 2.50).

Regarding chronic disease progression and lifelong medication, practicing PAs showed the highest agreement (mean = 3.25) compared to other groups (means ranging from 2.47 to 2.89). Views on food additives and processing showed a consistent trend toward viewing them as significant health factors, with PA students expressing the strongest concern (mean = 1.49) compared to other groups (means ranging from 1.76 to 2.00). The use of biomarkers as management indicators received similar ratings across groups (means ranging from 1.50 to 2.23), as did perspectives on diabetes remission without medication (means ranging from 2.55 to 3.00). Notably, all groups showed a strong preference for lifestyle interventions over medication (means ranging from 1.74 to 2.00), indicating consistent alignment regardless of professional role.

Views on work hours versus sleep (means ranging from 2.09 to 2.38) and confidence in medical education's ability to incorporate new research (means ranging from 2.30-to 2.75) showed minimal variation across groups, suggesting shared perspectives on these systemic issues regardless of career stage or professional role.

## Discussion

The patterns observed in this cross-sectional survey reveal both systemic constraints and their consequences in the evolution of medical education. A noticeable finding was the 100% exclusive use of written, video, or audio content by practicing healthcare professionals, contrasting with students and residents who accessed more diverse knowledge sources, including peers, mentors, and faculty. This transition might indicate a professional plateau, reduced access to mentorship, or simply the practical constraints of clinical practice prioritizing easily accessible resources. While students show some diversity in knowledge sources, with nearly half of PA students and a third of medical students citing faculty as primary resources, these faculty members themselves rely predominantly on standardized content. This creates a closed loop where even apparent diversity in knowledge sources ultimately channels back to the same foundational materials, reinforcing rather than challenging existing paradigms.

The socio-ecological model provides a framework for understanding how this system self-perpetuates. The demands of academic testing, which require increasingly detailed rote memorization, create a system where exploration of alternative perspectives becomes a luxury few can afford. Students face a Hobson’s choice: dedicate their limited time to mastering the "correct" answers required for academic success or risk their professional advancement by questioning established paradigms. This educational inertia manifests clearly in the paradoxical beliefs about chronic disease management. Our results showed noteworthy consistency across professional identities in simultaneously endorsing biomarkers as valid indicators, expressing skepticism about medication-free remission, yet believing strongly in the superiority of lifestyle interventions. This cognitive dissonance is particularly noticeable when considering the publication of many studies [4-7], which demonstrate successful medication-free diabetes reversal. The reliance on predominantly medication-controlled metrics as primary indicators of success, while acknowledging lifestyle interventions' superiority, reveals the gap between evidence and practice that the current educational system struggles to bridge.

Our results suggest a similar troubling pattern regarding evidence-based medicine. Practitioners showed higher confidence in evidence-based guidelines despite extensive literature demonstrating their frequently low quality and potential bias [8-13]. As practicing physicians and advanced practice providers gain experience in the medical model, which promotes guideline-based medicine, it becomes harder and harder to accept that this method is erroneous. This cognitive dissonance may get primed from the standardized testing environment that reinforces the concept of singular, infallible truths. The motivation to succeed academically may inadvertently create resistance to questioning established paradigms throughout career progression.

The perpetuation of these beliefs is reinforced by a "rite of passage" mentality in medical and PA education. After investing countless hours in studying, testing, and clinical training that emphasizes standardized knowledge, practitioners may find it difficult to question whether this intensive preparation translates to optimal clinical outcomes. This bias extends to beliefs about sleep deprivation in medical training despite overwhelming evidence of its detrimental effects on performance [14-22]; those who have endured it often defend its necessity, perhaps as a way of validating their own experience.

The relentless progression from education to career creates a self-reinforcing cycle. The anxiety-filled rush to master accepted models during training transitions seamlessly into the pressures of clinical practice, leaving little space for questioning fundamental assumptions. The time and mental energy required to master and operate within the current system effectively preclude the exploration of alternative paradigms, even when evidence supporting such alternatives exists.

While the current system successfully produces competent professionals, our findings suggest the need for thoughtful evolution in medical and PA education. This might involve creating intentional space for critical thinking throughout the educational process, restructuring evaluation methods to reward innovation alongside knowledge recall, and explicitly acknowledging the potential limitations of current paradigms. By recognizing these systemic constraints while leveraging existing strengths, medical and PA education could evolve to better balance standardization with innovation, ultimately creating an increasingly effective and adaptable healthcare system.

Limitations

Several important limitations should be considered when interpreting our findings. First, the single-institution design, while allowing for controlled examination of belief transmission within one medical ecosystem, limits generalizability to other academic medical centers or healthcare settings. Cultural and regional factors specific to Oklahoma may influence beliefs and practices in ways that differ from other geographical or institutional contexts.

The cross-sectional nature of our study captures beliefs at a single point in time, preventing analysis of how individual beliefs evolve throughout professional development. A longitudinal study following medical and PA students through their training and into practice would better elucidate the mechanisms of belief formation and transformation. Our sample size, particularly among PA faculty, limits statistical power for some comparisons. The relatively small number of participants in certain categories may have prevented the detection of meaningful differences between groups. The lack of significant differences in the subgroup analyses may suggest a uniformity of beliefs across professional roles, which aligns with the concept of entrenched paradigms being pervasive throughout the medical community. Alternatively, this finding may be influenced by the sample size or composition, potentially limiting the ability to detect subtle differences. Future studies with larger, more diverse samples may provide greater insights into subgroup variations. Furthermore, response bias may have influenced our results, as individuals with stronger opinions about medical education and practice might have been more likely to participate.

The survey instrument, while carefully developed, may not fully capture the complexity and nuance of medical beliefs. The use of Likert scales, while facilitating quantitative analysis, necessarily reduces complex viewpoints to simplified responses. Additionally, despite efforts to minimize social desirability bias through anonymous collection, participants may have provided responses they believed to be professionally acceptable rather than reflecting their true beliefs.

Future considerations​​​​​​​

To build upon our findings, future research should consider conducting multi-institutional studies involving a diverse range of medical schools and healthcare institutions. Such studies would enhance the generalizability of the results and allow for the examination of how different educational environments impact the persistence of entrenched beliefs. Increasing the sample size is also crucial. Larger studies would provide greater statistical power to detect differences between subgroups and strengthen the validity of the conclusions drawn.

Employing mixed-methods approaches could offer a more comprehensive understanding of the issue. Combining quantitative surveys with qualitative methods, such as interviews or focus groups, would enable researchers to explore the reasons behind clinicians' adherence to certain beliefs. This could uncover cultural, educational, or experiential factors that quantitative data alone might not reveal. Additionally, longitudinal studies tracking changes in beliefs over time and in response to specific educational interventions could provide valuable insights into the effectiveness of strategies aimed at fostering critical thinking and adaptability in medical education.

## Conclusions

This pilot study suggests a potential misalignment between current medical education approaches and population health outcomes in the United States, where, despite advances in medical knowledge and increasing numbers of healthcare graduates, population health metrics continue to decline. While medical and PA education have evolved in their teaching methodologies, these changes have primarily focused on optimizing the delivery of standardized information rather than fundamentally reconsidering what we teach. Our findings indicate consistent patterns across professional levels in emphasizing medicalization and pharmaceutical interventions while potentially undervaluing critical thinking and alternative approaches to health and healing.

Though standardized knowledge forms a necessary foundation for medical practice, our results suggest that the current emphasis on rote memorization may come at the expense of developing critical thinking skills. Students sacrifice personal, emotional, and physical well-being to master standardized content that, once internalized, becomes increasingly resistant to challenge or modification. While these preliminary results require validation through more comprehensive research, they point to opportunities for examining how medical and PA education might be restructured to better balance standardized knowledge acquisition with the development of adaptive thinking skills that could help practitioners evaluate and integrate emerging evidence throughout their careers.

## References

[1] Bourdieu P, Passeron J-C (1990). Reproduction in education, society and culture. Sage.

[2] Hafferty FW (1998). Beyond curriculum reform: confronting medicine's hidden curriculum. Academic Medicine.

[3] Hodges BD (2010). A tea-steeping or i-Doc model for medical education?. Acad Med.

[4] Athinarayanan SJ, Adams RN, Hallberg SJ (2019). Long-term effects of a novel continuous remote care intervention including nutritional ketosis for the management of type 2 diabetes: a 2-year non-randomized clinical trial. Front Endocrinol (Lausanne).

[5] Hussain TA, Mathew TC, Dashti AA, Asfar S, Al-Zaid N, Dashti HM (2012). Effect of low-calorie versus low-carbohydrate ketogenic diet in type 2 diabetes. Nutrition.

[6] Moriconi E, Camajani E, Fabbri A, Lenzi A, Caprio M (2021). Very-low-calorie ketogenic diet as a safe and valuable tool for long-term glycemic management in patients with obesity and type 2 diabetes. Nutrients.

[7] Rafiullah M, Musambil M, David SK (2022). Effect of a very low-carbohydrate ketogenic diet vs recommended diets in patients with type 2 diabetes: a meta-analysis. Nutr Rev.

[8] Astrup A, Teicholz N, Magkos F (2021). Dietary saturated fats and health: are the US guidelines evidence-based?. Nutrients.

[9] Davis MS, Riske-Morris M, Diaz SR (2007). Causal factors implicated in research misconduct: evidence from ORI case files. Sci Eng Ethics.

[10] Every-Palmer S, Howick J (2014). How evidence-based medicine is failing due to biased trials and selective publication. J Eval Clin Pract.

[11] Fanaroff AC, Califf RM, Windecker S, Smith SC Jr, Lopes RD (2019). Levels of evidence supporting American College of Cardiology/American Heart Association and European Society of Cardiology Guidelines, 2008-2018. JAMA.

[12] Guerra-Farfan E, Garcia-Sanchez Y, Jornet-Gibert M, Nuñez JH, Balaguer-Castro M, Madden K (2023). Clinical practice guidelines: the good, the bad, and the ugly. Injury.

[13] Torgerson T, Wayant C, Cosgrove L (2022). Ten years later: a review of the US 2009 institute of medicine report on conflicts of interest and solutions for further reform. BMJ Evid Based Med.

[14] Garbarino S, Lanteri P, Bragazzi NL, Magnavita N, Scoditti E (2021). Role of sleep deprivation in immune-related disease risk and outcomes. Commun Biol.

[15] Kancherla BS, Upender R, Collen JF (2020). Sleep, fatigue and burnout among physicians: an American Academy of Sleep Medicine position statement. J Clin Sleep Med.

[16] Krause AJ, Prather AA, Wager TD, Lindquist MA, Walker MP (2019). The pain of sleep loss: a brain characterization in humans. J Neurosci.

[17] Krause AJ, Simon EB, Mander BA, Greer SM, Saletin JM, Goldstein-Piekarski AN, Walker MP (2017). The sleep-deprived human brain. Nat Rev Neurosci.

[18] Kroshus E, Wagner J, Wyrick D (2019). Wake up call for collegiate athlete sleep: narrative review and consensus recommendations from the NCAA Interassociation Task Force on Sleep and Wellness. Br J Sports Med.

[19] Lenzer J (2015). Doctors underwent "extreme sleep deprivation" in studies of effect on patient deaths. BMJ.

[20] Thompson KI, Chau M, Lorenzetti MS, Hill LD, Fins AI, Tartar JL (2022). Acute sleep deprivation disrupts emotion, cognition, inflammation, and cortisol in young healthy adults. Front Behav Neurosci.

[21] Venkatraman V, Chuah YM, Huettel SA, Chee MW (2007). Sleep deprivation elevates expectation of gains and attenuates response to losses following risky decisions. Sleep.

[22] Walker MP (2008). Cognitive consequences of sleep and sleep loss. Sleep Medicine.

